# Innovative Assistive Technologies for Tetraplegia: A Narrative Review of Systematic and Emerging Evidence

**DOI:** 10.3390/healthcare14020274

**Published:** 2026-01-21

**Authors:** Lorenzo Desideri, Regina Gregori Grgič, Antonia Pirrera, Daniele Giansanti

**Affiliations:** 1 Digital Psychology Lab, Sigmund Freud University, 20143 Milano, Italy; l.desideri@milano-sfu.it (L.D.); r.gregori@milano-sfu.it (R.G.G.); 2 National Center IATIS, Istituto Superiore di Sanità-Roma, 00161 Roma, Italy; antonia.pirrera@iss.it

**Keywords:** assistive technology, spinal cord injury, tetraplegia, quadriplegia

## Abstract

**Background:** Assistive technologies (ATs) for individuals with tetraplegia have evolved from mechanical aids to complex neurotechnological, digital, and psychosocial systems. However, the evidence base remains fragmented, with heterogeneous methodologies and limited integration across domains. This review synthesizes recent review-level evidence to clarify current trends, gaps, and directions in ATs for tetraplegia. **Methods:** A narrative review of reviews was conducted following the ANDJ checklist. PubMed and Scopus were searched for systematic, scoping, and narrative reviews addressing assistive technologies relevant to tetraplegia. After screening, de-duplication, and quality appraisal, 20 reviews were included and synthesized narratively. **Results:** The included reviews clustered into four main domains: neural and regenerative interfaces, motor and biomechanical assistive systems, digital and adaptive rehabilitation ecosystems, and psychosocial and integrative frameworks. Across domains, evidence highlights a shift toward personalized, adaptive, and interoperable systems, supported by neurotechnologies, robotics, mobile health, and virtual reality. Common limitations include heterogeneous outcome measures, scarcity of longitudinal evidence, limited system interoperability, and persistent inequities in access and adoption. Emerging applications of artificial intelligence support adaptive control, monitoring, and personalization, though robust clinical validation remains limited. **Conclusions:** This synthesis provides a structured overview of review-level evidence on assistive technologies for tetraplegia. The findings underscore the need for coordinated, multidisciplinary approaches and more rigorous, longitudinal evaluation to support the development of inclusive, human-centered, and interoperable assistive ecosystems.

## 1. Introduction

### 1.1. Clinical and Social Context

Tetraplegia, most commonly caused by a cervical spinal cord lesion, represents one of the most severe forms of physical disability worldwide. It involves a partial or complete loss of voluntary motor and sensory functions in all four limbs, often accompanied by trunk and respiratory impairment [1,2]. Beyond paralysis, individuals frequently experience secondary complications such as respiratory dysfunction, spasticity, chronic pain, and autonomic dysregulation, resulting in a complex and lifelong clinical condition [3].

Tetraplegia, also referred to as quadriplegia, represents the most severe form of spinal cord injury (SCI). It occurs when lesions affect the cervical spinal segments (C1–C8), resulting in significant impairments of both upper and lower limbs, trunk stability, and, in higher lesions, respiratory function [4,5,6,7]. The precise level of the lesion strongly influences the degree of functional loss: injuries at higher cervical levels (C1–C4) can compromise both arm and respiratory muscles, often necessitating ventilatory support, whereas lower cervical injuries (C5–C8) may preserve some arm and hand function but still result in substantial limitations in mobility and daily activities.

In contrast, paraplegia arises from lesions below the cervical level, typically in the thoracic, lumbar, or sacral segments, and primarily affects the lower limbs while sparing upper-limb function. As a result, individuals with tetraplegia generally experience greater dependence in activities of daily living, mobility, and communication compared with those with paraplegia [4,8]. Despite these differences, both tetraplegia and paraplegia share common challenges related to sensory-motor loss, secondary health complications, and the need for assistive technologies, though the severity and distribution of functional impairments differ substantially.

Beyond clinical severity, tetraplegia has far-reaching psychosocial and societal consequences. Individuals frequently experience reduced participation in education, employment, and community life, while caregivers and family members assume substantial long-term emotional and practical burdens [9]. High rates of anxiety, depression, social isolation, and diminished quality of life are consistently reported [10]. Cervical injuries are also associated with life-threatening complications such as ventilatory insufficiency, autonomic dysreflexia, and cardiovascular instability, contributing to increased mortality and reduced life expectancy compared with lower-level injuries [8].

Taken together, tetraplegia constitutes a persistent biopsychosocial challenge requiring highly individualized, multidisciplinary, and person-centred approaches that integrate medical, technological, and psychosocial perspectives.

### 1.2. Assistive Technology in Tetraplegia

Over recent decades, advances in acute care, physiotherapy, and neurorehabilitation have progressively shifted the clinical focus from survival toward independence. Within this evolving paradigm, assistive technologies (ATs) play a central role by bridging biological limitations and environmental demands, enabling communication, mobility, and interaction.

ATs encompass a broad range of devices, equipment, and systems designed to maintain or improve functional capabilities in individuals with physical, sensory, or cognitive impairments. According to the World Health Organization (WHO), ATs enhance mobility, communication, personal care, and participation in daily life by reducing environmental and functional barriers [11]. Their scope includes both hardware—such as wheelchairs, prosthetics, and environmental control systems—and software solutions, including speech-generating applications and adaptive computer interfaces, as highlighted by the Assistive Technology Industry Association (ATIA) [12]. Federal definitions, such as those from the ECTA Center, similarly describe ATs as any item or system used to increase, maintain, or improve functional capabilities [13].

For individuals with tetraplegia, ATs play a crucial role in supporting mobility, communication, self-care, and daily living [11,12,13]. Mobility aids such as powered wheelchairs, upper-limb exoskeletons, and standing devices enhance independence, reduce reliance on caregivers, and facilitate participation in social and professional contexts [14,15]. Communication technologies—including speech-generating devices, eye-tracking systems, head-controlled interfaces, and brain–computer interfaces—enable interaction, self-expression, and sustained social engagement across educational, occupational, and community settings [16,17].

Adaptive tools for eating and dressing, environmental control systems, and home automation further support autonomy in daily activities and improve quality of life [18]. Collectively, these technologies increasingly redefine autonomy in tetraplegia, not only by restoring function but also by enabling participation, self-expression, and social belonging [14,15,16,17,18].

### 1.3. Rationale and Objectives

#### 1.3.1. Rationale for the Study

SCI, and particularly tetraplegia, is characterized by highly individualized patterns of functional limitation. Within the framework of the International Classification of Functioning, Disability and Health (ICF) (ICF link), outcomes can be systematically described across body functions, activities, and participation, providing a structured lens to assess the impact of assistive technologies on arm and hand function, mobility, communication, and social participation. Residual capacities vary widely depending on lesion level, severity, secondary complications, and personal and environmental factors, underscoring the need for personalized rehabilitation strategies and targeted technological support.

Despite their potential to enhance autonomy and participation, research on assistive technologies for tetraplegia remains fragmented across study designs, populations, and outcome measures. This heterogeneity limits comparability, obscures patterns of effectiveness, and complicates translation into clinical practice and policy.

For these reasons, this work is intentionally designed as a narrative review of reviews, synthesizing evidence from systematic reviews, meta-analyses, scoping reviews, and other review-level contributions to provide an integrative, higher-level perspective on assistive technologies in tetraplegia. The aim is not to pool primary evidence or generate quantitative effect estimates, but to consolidate existing syntheses, clarify conceptual and methodological trends, and identify converging themes, gaps, and future directions across the multidisciplinary literature.

#### 1.3.2. Aim of the Review

Given the expanding and diverse body of literature on assistive technologies for tetraplegia, a narrative review of reviews is warranted to synthesize the state of the art while preserving conceptual flexibility. Systematic and meta-analytic reviews provide methodological rigor but may overlook broader experiential and contextual dimensions, while scoping reviews offer breadth with variable critical depth. A narrative synthesis allows these perspectives to be integrated, highlighting patterns, divergences, and emerging directions.

Specifically, the review aims to map research trends by documenting the evolution of review-level literature on assistive technologies for tetraplegia and identifying emerging technological domains; to synthesize key themes across mobility, communication, and daily living support, with attention to autonomy and participation; and to identify clinical and societal implications, highlighting opportunities for translation into practice and policy, as well as gaps requiring further investigation.

## 2. Methods

Given the multidisciplinary complexity and evolving nature of AT for tetraplegia—characterized by diverse methodologies, terminological inconsistencies, and emerging intervention research—the narrative review methodology was selected for its flexibility, rigor, and practical applicability [19].

This approach allows integration of insights from a wide range of review types, including systematic, scoping, non-systematic, and meta-analytic reviews, capturing the breadth and diversity of research on AT while enabling synthesis of recurring patterns, methodological gaps, emerging trends, and contextual factors influencing adoption and outcomes across clinical, technological, and social domains.

By concentrating on reviews rather than individual primary studies, this methodology allows identification of consistent trends across different populations and study designs, highlights divergences in findings or methodological approaches, and uncovers knowledge gaps that may hinder the development of integrated, user-centered interventions [20]. It also facilitates synthesis of information across multiple contexts and disciplines, providing a comprehensive overview of how AT supports autonomy, social participation, and quality of life for individuals with tetraplegia.

### 2.1. Narrative Review of Reviews Approach, Search Strategy, and Quality Assessment

This narrative review was conducted following the principles of transparency and methodological consistency outlined in the ANDJ Narrative Review Checklist [21] and a consolidated structured quality control procedure also recalled in [22]. The aim was to synthesize high-quality evidence on assistive technologies (ATs) for individuals with tetraplegia, focusing on functional outcomes, usability, clinical implementation, and psychosocial impact, rather than purely technical aspects.

#### 2.1.1. Search Strategy, Study Selection, and Scope

The literature search targeted reviews on AT for tetraplegia, prioritizing studies reporting clinical relevance, functional impact, or translational value. Searches were conducted in PubMed and Scopus, covering publications in English up to 30 October 2025. All types of reviews were considered, including systematic, scoping, narrative, and meta-analytic reviews, to capture the full breadth of evidence.

Multiple keyword combinations were used to ensure comprehensive coverage of the relevant literature and to facilitate discoverability for readers and researchers. “Tetraplegia” and “quadriplegia” are included because they are synonymous terms widely used in the literature to describe injuries affecting the cervical spinal cord, and using both ensures that studies employing either terminology are captured. “Spinal cord injury (SCI)” is included to encompass the broader population and to account for studies that report on mixed cohorts, where tetraplegia may be a subset.

Keywords related to interventions, such as “assistive technology” and “AAC” (augmentative and alternative communication), were selected to reflect the focus on solutions that support function, independence, and participation in daily life. Finally, domain-specific terms—covering mobility, communication, upper-limb function, and activities of daily living—ensure that the search captures studies addressing the key functional areas affected by cervical SCI. Together, these keywords maximize the likelihood of retrieving relevant evidence across clinical, technological, and functional domains, while maintaining precision in identifying literature directly relevant to tetraplegia.

The objective of this narrative synthesis was not to count primary studies or generate quantitative estimates, but to identify key themes, recurring patterns, clinical insights, and gaps, highlighting the contribution of different review types to the understanding of AT for tetraplegia.

The categorization presented in Table 1 organizes AT into focus areas to structure the literature and facilitate synthesis.

#### 2.1.2. Selection and Qualification of Reviews

The selection and qualification of reviews followed a structured multi-step process designed to ensure methodological rigor, clinical relevance, and transparency. All types of reviews—systematic, scoping, narrative, and meta-analytic—focusing on assistive technologies (ATs) for tetraplegia were considered, provided they reported functional outcomes, usability, or clinical application. Reviews covering broader spinal cord injury populations were included only if tetraplegia-specific results could be clearly extracted.

Titles and abstracts were screened independently and in a blinded manner by two reviewers using Rayyan. Any disagreements between reviewers were resolved through discussion and consensus, ensuring reliability and consistency in the screening process.

Each study that passed the initial screening underwent a detailed quality assessment based on six parameters: clarity of rationale, appropriateness of research design, transparency of methodology, presentation of results, validity of conclusions, and disclosure of potential conflicts of interest. Parameters N1 through N5 were scored on a 1-to-5 scale, while N6 was assessed as Yes/No. Only studies meeting the required thresholds across all six parameters for both reviewers were included in the final synthesis.

Data from the included reviews were then organized according to functional domains and conceptual focus. This approach allowed identification of recurring themes, emerging patterns, evidence gaps, and notable clinical or technological innovations. The resulting synthesis emphasized the integration of clinical, technological, and psychosocial dimensions, while also considering potential applications of artificial intelligence in assistive technologies for tetraplegia.

### 2.2. Screening Team and Reliability Assessment

The screening and selection of studies were conducted with a focus on methodological rigor, clinical relevance, and transparency. Two reviewers (A.P. and D.G.) independently screened titles and abstracts in a blinded manner using Rayyan and extracted data from full texts. Disagreements were resolved by discussion and consensus.

All records were imported into Rayyan for deduplication, independent screening, and application of inclusion/exclusion criteria (Table 2). Studies passing initial screening were assessed on the six parameters (N1–N6) as described in Supplementary Materials. N1–N5 were scored 1–5, and N6 was Yes/No. Only studies meeting all thresholds for both reviewers were included in the narrative synthesis, ensuring methodological consistency, transparency, and reliability.

### 2.3. Narrative Review of Reviews: Approach and Rationale

Given the multidisciplinary complexity and evolving nature of assistive technologies (ATs) for tetraplegia—characterized by diverse methodologies, terminological inconsistencies, and emerging intervention research—a narrative review of reviews was selected for its flexibility, rigor, and practical applicability [19,20].

This approach allows integration of insights from a wide spectrum of review types, including systematic, scoping, non-systematic, and meta-analytic reviews. It captures the breadth and diversity of research on AT, while enabling the synthesis of recurring patterns, methodological gaps, emerging trends, and contextual factors influencing adoption and outcomes across clinical, technological, and social domains.

Compared with a strictly systematic review, the narrative review offers several advantages:

1. Flexibility in handling heterogeneous evidence—it can accommodate varying study designs, intervention types, populations, and outcome measures, which are common in AT research.
2. Integration across disciplinary boundaries—it allows clinical, technological, psychosocial, and policy-relevant perspectives to be combined.
3. Identification of knowledge gaps and opportunities—by synthesizing trends and themes at the review level, it provides a foundation for highlighting areas where further research, innovation, or guideline development is needed.
4. Support for translational interpretation—findings from the narrative review can inform practical recommendations and contextualize how review-level conclusions relate to real-world practice and policy, which is more challenging in rigid systematic approaches [19,20].

Specifically, the narrative review provides the methodological foundation to systematically report:

- Research trends: evolution of review-level literature over time, including growth in publications and the emergence of novel technological domains.
- Thematic categorizations: organization of AT research into key functional domains such as mobility, communication, daily living, and psychosocial support.
- Gaps and recommendations: synthesis of areas where evidence is limited, inconsistent, or methodologically weak, as well as proposed directions for future research and clinical practice.

Importantly, while the narrative review identifies gaps and recommendations at the review level, it does not aim to quantitatively synthesize primary study outcomes. This preserves the focus on trends, themes, and opportunities emerging from secondary literature, providing a structured basis for subsequent interpretation in the Discussion.

### 2.4. Linking Review Outputs to Discussion

The Discussion section builds directly on the outputs of the narrative review of reviews, without introducing an independent analytical framework. Its purpose is to examine how the gaps and recommendations identified at the review level are being addressed—or remain unaddressed—within current primary research, international guidelines, and policy frameworks.

- Primary studies are used to illustrate emerging responses to identified gaps, such as improvements in usability, personalization, long-term effectiveness, and integration of psychosocial support. Their role is illustrative and contextual, not evidentiary.
- International guidelines, consensus statements, and policy documents are analyzed to evaluate the degree to which review-level recommendations are translated into clinical practice and health-system strategies. This includes aspects such as user-centered design, accessibility, ethical governance, and equitable deployment.

By structuring the Discussion around review-identified gaps and recommendations, this approach ensures continuity between aims, results, and interpretation. It also demonstrates the added value of a narrative review of reviews: the ability to move beyond isolated findings and situate emerging evidence, clinical practices, and guidelines within a coherent developmental trajectory of AT for tetraplegia.

Notably, this integrative and interpretative step would be difficult within a strictly systematic review framework, which—while essential for quantitative synthesis—offers limited flexibility to connect heterogeneous evidence sources, evolving practices, and translational implications across disciplinary boundaries [19,20].

## 3. Results

Building on the systematic selection and synthesis of 20 review studies, this section delineates the evolving scientific landscape of AT for tetraplegia.

Section 3.1 outlines the methodological framework used for study selection and inclusion criteria. It specifies how the evidence base was filtered and categorized, ensuring transparency and consistency across sources.

Section 3.2 situates the field within broader publication trends, tracing its historical development and evolving focus. It charts the growth of AT research for tetraplegia, from early mechanical solutions to the current integration of robotics, artificial intelligence, and neural interfaces.

Section 3.3 distils the main messages, recurring patterns, and thematic intersections that emerge across the reviewed literature. It highlights the converging emphasis on personalization, usability, and ethical responsibility, while also drawing attention to persisting gaps in long-term assessment, real-world adoption, and user engagement.

Section 3.4 synthesizes the emerging opportunities, challenges, and recommendations identified in the literature. It summarizes how collaborative, multidisciplinary, and policy-aligned approaches can accelerate translation from laboratory research to clinical and everyday application.

### 3.1. Study Selection

Initially, 631 records were identified in PubMed and 951 in Scopus. After focusing specifically on review articles, which allow integration of prior findings while emphasizing the most recent and updated evidence, the pool was narrowed to 77 reviews from PubMed and 116 from Scopus. Subsequent screening removed duplicates across databases, yielding 83 unique reviews. The final inclusion of 20 studies was based on a rigorous full-text quality assessment using a standardized checklist and quantitative framework [21,22], which also considered methodological transparency, reporting of prior work, and relevance to functional outcomes, discrepancies between the two reviewers were resolved through discussion, and consensus was always reached. This approach ensured that the selected reviews reflect the most current and high-priority evidence on assistive technologies for tetraplegia, providing a solid foundation for synthesis.

### 3.2. Biomedical Publication Trends

To frame research trends, two targeted PubMed searches were conducted using the keywords outlined in Box 1: one focusing on tetraplegia and assistive technologies (ATs), and the other on ATs more broadly. The tetraplegia-focused search includes both studies specifically addressing tetraplegia and quadriplegia, as well as studies indexed under the broader term spinal cord injury (SCI), capturing research on cervical and thoracic injuries (i.e., both tetraplegia and paraplegia). This approach allows comparison between publications highly specific to tetraplegia and those covering the wider AT field.

Interestingly, both searches trace back to one of the earliest studies on AT, a 1953 paper on quadriplegia [23]. Blau, Phillips, and Rose introduced a device aimed at enhancing self-sufficiency in individuals with quadriplegia, marking the beginning of scientific exploration in this area [20]. This highlights how early AT research was closely linked to improving life for individuals with tetraplegia.

For the tetraplegia + SCI subset (Table 3), 544 publications were identified, including 74 reviews (≈13.6%). The past decade alone contributed 343 studies (≈63%), with 187 (≈34%) published in the last five years. These works largely focus on mobility, upper-limb function, communication, and daily living, reflecting ongoing translation from research to practical interventions for individuals with cervical spinal cord injuries.

In contrast, the broader AT field has expanded substantially, with 8650 publications identified, including 1288 reviews (≈14.9%). The last decade contributed 5942 studies (≈68.7%), with 3789 (≈43.8%) published in the last five years. This growth reflects increasing global interest in assistive solutions across a wide spectrum of disabilities, and advances in technology from mobility aids to brain–computer interfaces.

These trends show that tetraplegia + SCI research is a specialized subset of the AT literature, reflecting the complex functional limitations and rehabilitation needs of individuals with cervical injuries. Review articles indicate ongoing efforts to synthesize knowledge and guide clinical practice and technology development. The recent surge in publications across both domains highlights a convergence of clinical need and technological capability, promoting integrated solutions that support mobility, communication, and daily living.

Box 1 **The used search keys.**

((tetraplegia[Title/Abstract]) OR (tetraplegic*[Title/Abstract]) OR (quadriplegic*[Title/Abstract]) OR (“spinal cord injury”[Title/Abstract]) OR (quadriplegia[Title/Abstract])) AND (“Assistive tech*”[Title/Abstract] OR “Assistive dev*”[Title/Abstract])

(“Assistive tech*”[Title/Abstract] OR “Assistive dev*”[Title/Abstract])

### 3.3. Emerging Themes and Categorization

The initial overview of reviews considered 21 studies [24,25,26,27,28,29,30,31,32,33,34,35,36,37,38,39,40,41,42,43,44], of which 20 were included in the subsequent narrative analysis. Reference [29], although not formally included in the main synthesis, is highly relevant and noteworthy. It presents an innovative approach using microneedle patches for exosome delivery, which the authors describe as potentially applicable as an assistive product, demonstrating novel mechanisms that could complement assistive technology interventions in spinal cord injury. We highlight this study here to underscore its potential contribution to the broader understanding of therapeutic strategies and technological applications, even if it is not part of the core analysis.

#### 3.3.1. Emerging Themes

A synthesis of the 20 reviews included is presented in Table 4, outlining their main focus and proposed AT applications. Collectively, these works illustrate the multidimensional landscape of tetraplegia rehabilitation, bridging neurophysiological, biomechanical, psychosocial, and digital innovation domains.

Núñez Sardinha et al. [24] highlight the importance of user engagement and personalization for long-term adoption of assistive technologies. Mota et al. [25] describe a growing ecosystem of mobile health tools for SCI rehabilitation, noting gaps in clinical validation and interoperability. Elliott et al. [26] extend the field to sexual and reproductive health, emphasizing inclusive design and AT integration. Kang et al. [27] focus on wheelchair usability, stressing ergonomic optimization to enhance mobility and prevent shoulder injuries.

From a neurophysiological perspective, Stieglitz et al. [28] compare electrical stimulation paradigms after central paralysis and amputation, advocating individualized neuromodulation. De Pastina et al. [30] link sensorimotor remapping and peripersonal space reorganization to rehabilitation potential, connecting neuroplasticity with cognitive recovery.

Psychosocial insights are provided by Mitchell et al. [31], who identify autonomy and environmental support as key for AT integration at work, and by Gurung et al. [37], showing that sociostructural factors often outweigh device-centered interventions in improving quality of life. Morone et al. [35] synthesize these perspectives, mapping evolution, opportunities, and bottlenecks in clinical AT integration.

Cutting-edge neurotechnologies are represented by Sawyer et al. [32] and Colucci et al. [40], advocating BCI systems paired with robotic exoskeletons to restore autonomy, complemented by Khalid et al. [43] and Plaza et al. [44] on adaptive robotic rehabilitation, and Vibhuti et al. [42] on VR-based home training.

Biomechanical approaches include Arellano and Vega [33] on arm–leg coordination in gait rehabilitation, Fallahzadeh Abarghuei and Karimi [38] on orthotics via the ICF framework, and An et al. [34] on universally accessible exercise equipment. Novel interaction strategies are explored by Pirrera et al. [39] with the tongue barbell interface.

Finally, Alazzam et al. [36] highlight metabolic monitoring post-SCI, and Onate-Figuérez et al. [41] link fatigue to physiological and psychological factors, advocating smarter, data-driven assistive systems. Overall, these studies converge on a multidimensional vision of AT—spanning neural, biomechanical, digital, and environmental domains—where personalization, interoperability, and inclusivity drive innovation and user empowerment.

#### 3.3.2. Categorization

To better interpret the diversity of approaches emerging in the recent literature, the selected studies can be organized into four overarching domains: Neural and Regenerative Interfaces, Motor and Biomechanical Assistive Systems, Digital and Adaptive Rehabilitation Ecosystems, and Psychosocial and Integrative Frameworks (Table 5). These four domains represent the dominant areas of research in AT for tetraplegia, capturing the main directions through which innovation and rehabilitation currently evolve. As expected, several studies span multiple domains, reflecting the intrinsic interconnection between neural, biomechanical, digital, and psychosocial dimensions of recovery.

Although each category reflects a specific entry point into AT for tetraplegia, together they reveal a convergent effort toward restoring autonomy, embodiment, and participation through personalized, interoperable, and inclusive innovations.

Neural and Regenerative Interfaces encompass studies exploring brain–computer interfaces, electrical stimulation, and neuroplastic mechanisms for functional recovery [28,30,32,40]. These works emphasize the transition from compensatory devices to restorative paradigms, where the nervous system itself becomes part of the assistive loop. In particular, exosome-based and bioengineered delivery systems ù illustrate how molecular and cellular approaches can complement neural engineering to promote long-term functional repair.

Motor and Biomechanical Assistive Systems include interventions targeting posture, mobility, and movement coordination [27,33,34,38,43,44]. Here, the focus shifts to ergonomic optimization and hybrid mechanical–neural control, aiming to reduce fatigue, prevent secondary injuries, and enable active participation in daily activities.

Digital and Adaptive Rehabilitation Ecosystems cover the rapidly expanding domain of mobile health, tele-rehabilitation, and virtual reality [25,35,42]. These studies highlight the potential of data-driven feedback, gamified engagement, and home-based interventions, though gaps remain in interoperability and evidence-based validation.

Finally, Psychosocial and Integrative Frameworks [24,26,31,36,37,39,41] stress that meaningful recovery extends beyond physical function. By addressing quality of life, sexuality, self-efficacy, and sociostructural determinants, they remind us that assistive technologies succeed only when embedded within inclusive and supportive ecosystems.

Together, these domains outline a multidimensional roadmap for innovation in tetraplegia, where the boundary between body, device, and environment progressively dissolves into a dynamic human–technology continuum.

To further clarify the diversity of assistive technologies, a conceptual distinction can be outlined between invasive vs. non-invasive, robotic, digital, and AI-based systems. Neural and regenerative interfaces mainly include invasive neurotechnologies such as BCIs and electrical stimulation [28,30,32,40], whereas motor and biomechanical assistive systems are generally non-invasive, mechanical or hybrid robotic devices [27,33,34,38,43,44]. Digital and adaptive rehabilitation ecosystems encompass software-driven, AI-assisted, and connectivity-focused interventions [25,35,42], while psychosocial and integrative frameworks involve human-centered and environmental adaptations [24,26,31,36,37,39,41]. Some studies span multiple categories, reflecting the hybrid and interdisciplinary nature of contemporary assistive technologies. This distinction helps readers interpret the breadth of approaches and their potential interactions without implying rigid boundaries.

### 3.4. Emerging Opportunities, Challenges, Gaps, and Recommendations

Recent research highlights both promising opportunities and enduring challenges in the development and integration of assistive technologies for spinal cord injury, reflecting the dynamic convergence between clinical innovation and real-world application.

#### 3.4.1. Emerging Opportunities

Recent advances in assistive technologies for tetraplegia reveal a convergence between neural, biomechanical, digital, and psychosocial innovation domains. At the neurotechnological frontier, brain–computer interfaces and electrical stimulation systems are redefining the boundaries between compensation and restoration. Sawyer et al. [32] and Colucci et al. [40] demonstrated that BCI-controlled and hybrid exoskeletons can enable partial restoration of volitional control, while Stieglitz et al. [28] highlighted the growing personalization of electrical stimulation paradigms. De Pastina et al. [30] linked these mechanisms to peripersonal space remapping, suggesting new pathways for cognitive-motor integration.

In parallel, biomechanical and robotic assistive systems are expanding their scope from mobility support to active participation. Kang et al. [27] optimized manual wheelchair usability to minimize shoulder injuries, whereas Plaza et al. [44] and Khalid et al. [43] explored the adaptability of exoskeletons for lower and upper limbs. Arellano and Vega [33] illustrated how arm–leg coordination could enhance gait recovery, and Fallahzadeh Abarghuei and Karimi [38] confirmed the health benefits of tailored orthoses within the ICF framework.

Digital health ecosystems are also maturing rapidly. Mota et al. [25] described the proliferation of mobile health apps for spinal cord injury, while Vibhuti et al. [42] demonstrated how virtual reality can extend neuromotor rehabilitation into the home environment. Morone et al. [35] framed these trends within a broader narrative of technological integration, emphasizing interoperability as a precondition for sustainable clinical use.

From a human-centered perspective, inclusive and psychosocial innovation is gaining recognition. Núñez Sardinha et al. [24] revealed that acceptance and engagement are key determinants of long-term AT success, and Mitchell et al. [31] underlined the importance of workplace adaptation and autonomy. Elliott et al. [26] introduced sexual and reproductive health as an emerging dimension of rehabilitation, while Gurung et al. [37] and Onate-Figuérez et al. [41] identified fatigue and environmental factors as critical but modifiable determinants of quality of life. Finally, Pirrera et al. [39] and An et al. [34] opened new design avenues with non-traditional control systems (e.g., tongue interfaces) and universally accessible exercise devices.

Together, these studies depict an increasingly integrated ecosystem where neuroplasticity, robotics, digital connectivity, and user experience converge to enhance autonomy, embodiment, and participation in individuals with tetraplegia.

#### 3.4.2. Persistent Challenges

Despite these advances, major challenges still hinder the translation of assistive innovations into real-world impact. A recurring limitation is the fragmentation between technological and clinical validation pipelines. Many prototypes, such as mHealth tools [25] and VR-based interventions [42], demonstrate feasibility but lack standardized outcome measures or longitudinal evidence. Similarly, Sawyer et al. [32] and Colucci et al. [40] noted that invasive or hybrid BCI systems face ethical, regulatory, and usability barriers before they can transition from research to daily life.

Another barrier concerns interoperability and personalization. Studies by Núñez Sardinha et al. [24] and Morone et al. [35] showed that user acceptance often declines when technologies fail to adapt to the evolving physical and psychosocial needs of individuals. Kang et al. [27] and Plaza et al. [44] highlighted ergonomic mismatches and usability constraints that limit sustained engagement, particularly for upper-limb devices.

From a systemic view, inequities in access and contextual implementation remain pressing. Gurung et al. [37] identified social and environmental barriers that can outweigh technological efficacy, while Mitchell et al. [31] emphasized that supportive workplace environments are crucial for long-term integration. Even in highly technical domains, such as metabolic monitoring [36] or sexual health [26], the absence of tailored, inclusive frameworks perpetuates gaps in care.

Finally, the field faces a conceptual and ethical challenge: balancing innovation with user dignity and agency. The work by Pirrera et al. [39] on tongue-barbell control, while ingenious, exemplifies the tension between invasiveness and autonomy. The same ethical reflections extend to implanted BCIs and data-driven rehabilitation systems, where privacy, consent, and identity become integral to design.

Altogether, the literature suggests that the next frontier will depend not solely on technological sophistication, but on the co-design of interoperable, evidence-based, and ethically sustainable assistive ecosystems.

#### 3.4.3. Gaps, Emerging Trends, and Recommendations

Beyond identifying persistent challenges, it becomes essential to translate them into actionable directions for research and practice. Building on the opportunities and challenges discussed above, recent research reveals a set of converging gaps and transformative directions for future development. Across all domains—neural, biomechanical, digital, and psychosocial—three overarching needs emerge: methodological robustness, system interoperability, and genuinely human-centered design (Table 6).

First, methodological gaps persist in the generation of clinical evidence. Many studies still rely on short-term or small-sample designs, limiting external validity and real-life transferability. Longitudinal, multicenter trials are required to evaluate safety, durability, and adaptation over time—particularly for brain–computer interfaces [32,40] and mHealth or virtual-reality-based interventions [25,42]. The integration of AI-driven analytics could enhance monitoring and personalization, but this potential depends on transparent validation using diverse and representative datasets.

Second, interoperability remains a structural bottleneck. Current systems frequently operate in isolation: neural, mechanical, and digital layers rarely communicate through shared standards or modular frameworks. As highlighted by Morone et al. [35] and Kang et al. [27], advancing toward data-centric, context-aware ecosystems would enable more adaptive and sustainable assistive environments. Artificial intelligence could act as the connective layer across these heterogeneous components, supporting multimodal data fusion and intelligent control.

Third, psychosocial and ethical dimensions require deeper integration. Núñez Sardinha et al. [24] and Mitchell et al. [31] underscore that participatory design and co-evaluation foster acceptance, equity, and continuity of use. Meanwhile, evolving ethical frameworks must address data privacy, algorithmic transparency, and the delicate balance between autonomy and technological dependence.

Ultimately, the literature converges toward a shared vision: assistive technology for tetraplegia must transition from isolated devices to learning, interoperable ecosystems—adaptive, inclusive, and co-created with users. The next phase of progress will depend on bridging disciplinary silos and embedding artificial intelligence not as a replacement for human agency, but as its amplifier. Within this transformation, artificial intelligence emerges as a transversal enabler, bridging methodological, technical, and human-centered dimensions. Its role extends from adaptive signal processing to personalized rehabilitation and ethical data stewardship. The specific gaps, emerging trends, and recommendations—together with the cross-domain role of AI—are summarized in Table 6.

## 4. Discussion

Building on the integrated synthesis of the 20 reviewed studies—consisting of both systematic and non-systematic reviews—this discussion draws on the recommendations and gaps they identify to explore how subsequent research, including empirical investigations and policy-driven initiatives, has evolved in response. In doing so, it contextualizes the current state of knowledge on assistive technology for tetraplegia, highlighting both consolidated evidence and future directions for research and practice. Across neurotechnological, biomechanical, digital, and psychosocial domains, AT emerge as a critical enabler of autonomy, participation, and quality of life for individuals with tetraplegia.

Through this synthesis, key gaps and actionable recommendations naturally emerged, encompassing methodological robustness, ecosystem integration, user acceptance, ethical frameworks, equitable access, and psychosocial rehabilitation. These insights provide the foundation for structuring the discussion into interconnected sections, each linking evidence, translational mechanisms, and strategic implications for innovation and implementation.

The discussion is grounded both in the evidence-based gaps identified across the reviewed literature and in the corresponding recommendations, and is organized into seven interconnected Section 4.1, Section 4.2, Section 4.3, Section 4.4, Section 4.5, Section 4.6 and Section 4.7. This structure enables a comprehensive integration of global knowledge, empirical findings, and international policy frameworks, fostering a coherent understanding of how assistive technologies can evolve toward inclusive, ethical, and sustainable ecosystems for tetraplegia care.

Section 4.1 revisits the historical and conceptual foundations of AT in the context of tetraplegia, tracing their evolution from early rehabilitative devices of the 1950s to contemporary intelligent and networked systems. This perspective underscores the enduring importance of AT as both a technological and social innovation, capable of enhancing independence and participation. The section also highlights the added value of conducting a narrative review of reviews, stressing its capacity to integrate diverse strands of evidence, reveal cross-cutting trends, and to provide a strategic lens for future research and clinical translation.

Section 4.2 situates these scientific advances within the rapidly expanding global market for AT. Recent projections reveal robust growth driven by demographic shifts, increased prevalence of neurological conditions, and AI-enabled innovation. This economic dynamism underscores the strategic relevance of AT for health systems worldwide, while also highlighting regional disparities in access, production, and reimbursement models—factors that must be addressed to ensure equitable diffusion of technology for tetraplegia care.

Section 4.3 bridges methodological and translational gaps by integrating findings from recent clinical trials and pilot studies. These works demonstrate how advances in robotics, neural interfaces, and soft assistive systems are progressively addressing the limitations of earlier research. By mapping these studies to identified gaps—ranging from methodological rigor to ethical and psychosocial integration—this synthesis illustrates a growing shift toward interoperable, adaptive, and human-centered assistive ecosystems.

Section 4.4 expands the focus to policy and governance, illustrating how international and national frameworks are shaping the implementation of AT. Through an exemplificative sample—including WHO-GATE, WHO-MTS-SCI-E, ICF, ASIA, MHRA, EAA, CAN/ASC, and C-CPG—this part demonstrates a convergent movement toward standardization, inclusivity, and evidence-based rehabilitation. At the same time, it reveals persistent heterogeneity in regulatory and access models, reinforcing the need for harmonized global strategies that bridge ethics, equity, and operational effectiveness.

Section 4.5 explores the transformative role of artificial intelligence (AI) as a unifying force across domains of function and care. Within the ICF framework, AI acts as a catalyst for personalization, participation, and adaptive interaction, extending the human–technology relationship beyond compensation toward collaboration. Recent evidence highlights AI’s contributions to diagnosis, rehabilitation, communication, and environmental control, while calling for standardized datasets, ethical safeguards, and inclusive design to ensure that innovation remains transparent and equitable.

Section 4.6 reflects critically on the methodological scope and boundaries of this narrative review, acknowledging its focus on review-based synthesis and the inclusion of complementary empirical studies. It also emphasizes the translational relevance ensured through alignment with global frameworks and the inclusion of AI-driven and human-centered approaches. Future research should advance through systematic and scoping reviews focused on hybrid brain–computer interfaces, ethical design in AI, and longitudinal outcomes, thereby reinforcing evidence-based pathways toward sustainable independence and digital inclusion for individuals with tetraplegia.

Finally, Section 4.7 highlights that in clinical practice, assistive technologies for paraplegia should be implemented as personalized, interoperable, and user-centered solutions, addressing motor, cognitive, and social needs while ensuring ethical use and equitable access. For future research, emphasis should be placed on evaluating long-term effectiveness, integrating AI-driven and adaptive technologies, and developing inclusive assistive ecosystems that enhance autonomy, participation, and quality of life.

### 4.1. Importance, Added Value and Highlights of the Study

The significance of focusing on assistive technologies (ATs) in tetraplegia is considerable. ATs have roots in supporting motor disabilities more broadly, with clear relevance to tetraplegia since the early 1950s [45]. Early works either addressed issues directly relevant to tetraplegia or were applicable to individuals with severe cervical spinal cord injuries. For example, Newman and Jameson (1951) discussed devices supporting upper-limb and mobility needs [46], Zadrożny (1951) introduced “The Stabilizer” for shoulder exercise [47], and Treanor, Cole, and Dabato (1954) examined selective re-education and assistive devices, laying the groundwork for individualized rehabilitation strategies [48]. The landmark study by Blau, Phillips, and Rose (1953) [23] introduced a device specifically enhancing self-sufficiency in quadriplegics, establishing a foundation for decades of innovation in AT research.

Given this long history, a narrative review of reviews provides a powerful approach to synthesize evidence across multiple studies, identify overarching trends, and evaluate decades of progress. Unlike conventional systematic reviews focused on narrow questions, this approach integrates general and tetraplegia-specific perspectives, revealing patterns, gaps, and emerging priorities, and guiding future research, technology development, and clinical practice [45].

The added value lies in capturing the full spectrum of AT research—from neurotechnological innovations and robotic systems to digital health platforms and psychosocial interventions. This approach enables identification of cross-cutting trends and emerging themes that may be missed in isolated studies, and supports strategic guidance for research and clinical practice by summarizing methodological limitations, technological gaps, and areas of unmet need. It also enhances translational relevance, emphasizing real-world applicability and human-centered design.

Recent AT research highlights convergence of neurotechnological, biomechanical, digital, and psychosocial innovations to enhance autonomy, participation, and quality of life. Advances include BCI-controlled systems and personalized electrical stimulation [28,30,32,40], adaptive exoskeletons, optimized wheelchairs, and tailored orthoses [27,33,38,43,44], as well as digital platforms and VR-based interventions extending rehabilitation into home and community settings [25,35,42]. Human-centered innovations addressing workplace adaptations, engagement strategies, and social and sexual health demonstrate that technological success depends on user experience and context [24,26,31,34,37,39,41].

Despite progress, challenges remain. Translation to real-world applications is limited by fragmented technology and clinical validation, insufficient interoperability, and inadequate personalization to evolving user needs [24,27,32,35,40,44]. Socio-economic and environmental barriers further constrain equitable access, while ethical issues—particularly for invasive or data-driven systems—raise questions about autonomy, consent, and privacy [31,39]. Addressing these gaps requires moving toward cohesive, interoperable, and human-centered AT ecosystems, integrating insights from decades of research to support interventions that are technologically sophisticated, ethically grounded, and responsive to the complex needs of individuals with tetraplegia.

### 4.2. Global Market Trends and Growth Projections in Assistive Technologies: Implications for Tetraplegia Care

This section provides a contextual analysis of the global and tetraplegia-specific assistive technology (AT) market, aimed at framing the broader environment in which research, development, and clinical adoption occur. The data were collected from recent market reports and forecasts, focusing on growth trends, technological innovation, and regional dynamics.

Recent market analyses indicate robust growth in the global AT market, driven by demographic shifts, rising prevalence of disabilities, and rapid technological innovation. One report estimates the market at approximately USD 22.98 billion in 2023, projected to surpass USD 32.25 billion by 2030, with a compound annual growth rate (CAGR) of around 5% [49]. Another source forecasts growth from USD 26.8 billion in 2024 to about USD 41.0 billion by 2033, corresponding to a CAGR of 4.33% [50]. Additional analyses highlight an incremental increase of USD 6.3 billion between 2025 and 2029, driven by orthopedic and neurological disorders and the integration of AI in assistive devices (CAGR ~4.5%) [51].

For devices specific to tetraplegia, the global market is estimated at USD 6.4 billion in 2024, projected to reach USD 11.25 billion by 2032 (CAGR ~6.8%) [52]. A complementary forecast predicts growth from USD 3.2 billion in 2024 to USD 5.8 billion by 2034, with a CAGR of about 6.1% [53]. Regionally, North America’s assistive technology market is valued at USD 8.7 billion in 2023 and is expected to grow substantially by 2030, reflecting strong infrastructure, insurance coverage, and demand [54].

Mobility aids continue to dominate the product landscape, while the Asia Pacific region represents the fastest-growing segment. These trends reflect not only rising demand for mobility, communication, hearing, and vision support but also the expanding role of AT in digital health, AI-driven solutions, and consumer-oriented devices [51]. Overall, the market growth underscores the strategic importance of AT in healthcare and rehabilitation, highlighting that innovation, production, and reimbursement strategies will be key to sustaining this trajectory.

### 4.3. Integrating Recent Clinical Evidence to Address Gaps and Recommendations in Assistive Technology for Tetraplegia

In alignment with the gaps and recommendations outlined in Table 6, recent research over the past decade has increasingly focused on generating robust clinical evidence while integrating technological, psychosocial, and ethical dimensions in AT for tetraplegia. To maintain methodological consistency and comparability with the review-level synthesis, the same keyword-based search strategy applied in the narrative review of reviews was used to identify relevant studies. These recent studies, including clinical trials and RCTs, address methodological rigor, ecosystem integration, user acceptance, ethical considerations, access, and psychosocial rehabilitation, as summarized in Table 7. This integrative approach is essential to address the primary methodological gap (Gap 1, Table 6), namely the limited robustness of existing studies, which often rely on small samples, short follow-ups, and heterogeneous outcome measures, limiting comparability and generalization. Clinical trials and pilot studies, such as Lang et al. [55] and Bellicha et al. [56], demonstrate the safety, feasibility, and usability of powered exoskeletons and BCI-controlled robotics for mobility and daily activities, providing quantitative and longitudinal data that enhance methodological rigor. To overcome fragmentation of technological ecosystems (Gap 2, Table 6), studies such as de Crignis et al. [57] and Rubin et al. [58] show how integrating augmented reality, robotic arms, and neural interfaces facilitates interoperability among mechanical, neural, and digital components. Ting et al. [59] and Osuagwu et al. [60] further highlight the potential of sensor-based and soft-robotic devices for home-based rehabilitation, demonstrating feasibility for continuous, real-world use. User acceptance and long-term adherence (Gap 3, Table 6) are critical for AT success. Manns et al. [61] and Kong et al. [62] demonstrate that participatory design and co-evaluation methodologies, integrating user feedback iteratively, improve adherence and satisfaction. Similarly, Cappello et al. [63] and Sahadat et al. [64] show that adaptive, multimodal interfaces enhance functional outcomes and engagement, particularly for complex motor tasks. Ethical and regulatory uncertainties (Gap 4, Table 6), particularly for invasive devices like implanted BCIs [65,66] or tongue-controlled interfaces [62,64], underscore the importance of transparency, informed consent, privacy, and shared decision-making. Incorporating AI-based explainable and privacy-by-design systems ensures autonomy while maintaining ethical compliance. Gaps in equitable access and contextual adaptability (Gap 5, Table 6) and insufficient psychosocial integration (Gap 6, Table 6) are addressed in studies on home-based and interdisciplinary interventions. Osuagwu et al. [60], Ajiboye et al. [65], and Pandarinath et al. [66] highlight how AI-driven monitoring, adaptive device settings, and multimodal support enhance accessibility, rehabilitation, and social participation. Collectively, these studies show a shift from isolated devices to interoperable, adaptive, and human-centered assistive ecosystems. Leveraging insights from rigorous trials, AI-driven personalization, and participatory design, contemporary research bridges methodological, technical, and ethical gaps, ensuring AT for tetraplegia improves motor function, autonomy, psychosocial well-being, and sustainable adoption in real-world contexts.

### 4.4. Sample of International and National Guidelines on Assistive Technologies for Tetraplegia: Illustrative of Addressed Gaps and Recommendations

Focusing on both national and supranational guidance provides valuable insight into how the field of assistive technologies (ATs) for tetraplegia is evolving to address identified gaps and implement key recommendations. Although a full systematic review of existing guidelines lies beyond the scope of this study, a purposive sample of illustrative and contextual guidelines was selected to cover healthcare systems of global relevance, providing a comparative overview of how different contexts are responding to common challenges such as accessibility, equity, and clinical integration of AT (addressing Gap 5 in particular).

At the supranational level, the World Health Organization (WHO) has played a leading role in shaping both conceptual and operational frameworks. The Minimum Technical Standards and Recommendations for Spinal Cord Injury Management in Emergencies emphasizes the need for coordinated multidisciplinary care and minimum rehabilitation requirements—factors that directly impact the availability and proper use of assistive technologies in acute and post-acute phases, including for tetraplegia [70]. Complementarily, the WHO’s Global Cooperation on Assistive Technology (GATE) Initiative promotes global strategies to enhance access, affordability, and equity of AT devices, reinforcing a rights-based approach consistent with the UN Convention on the Rights of Persons with Disabilities (addressing Gaps 1, 4, and 5) [71].

At the national level, several high-impact frameworks from healthcare systems of relevance illustrate converging priorities and emerging differences:

- United States: Durable Medical Equipment Guide for Persons with Spinal Cord Injury or Dysfunction (ASIA, 2022) provides clinicians and end-users with detailed recommendations on device selection, training, and reimbursement pathways, fostering informed use and clinical continuity (Gaps 1 and 3) [72].
- United Kingdom: Assistive Technology: Definition and Safe Use guidance (MHRA, 2025) focuses on safety standards, interoperability, and user feedback mechanisms, aligning clinical efficacy with ethical and technical accountability (Gaps 2 and 4) [73].
- European Union: The European Accessibility Act (Directive 2019/882) establishes a regulatory framework for accessible products and services, including digital and assistive systems, promoting standardization and market harmonization (Gaps 2 and 5) [74].
- Canada: Accessibility Standards (CAN/ASC-EN 301 549:2024) [75] adapt the European framework to the North American context, reinforcing equitable access through user-centered design and inclusive procurement policies (Gaps 3 and 5).
- China: National clinical practice guidelines issued by the National Health Commission on Integrated Chinese and Western Medicine Rehabilitation for Spinal Cord Injury exemplify culturally contextualized rehabilitation practices, integrating traditional and modern approaches, emphasizing long-term recovery, social reintegration, and equitable distribution of assistive resources for individuals with tetraplegia (Gaps 5 and 6) [76].

Taken together, these selected documents demonstrate a shared global momentum toward standardization, inclusivity, and evidence-based rehabilitation, while also revealing persistent heterogeneity in implementation models and access mechanisms. This comparative overview is intended as a contextual sample, highlighting illustrative frameworks from key healthcare systems progressively addressing ethical and operational dimensions of AT for tetraplegia. A targeted future review mapping the interplay between clinical guidelines, assistive technologies, and long-term functional outcomes would be essential to consolidate this evolving field.

Table 8 reports a sketch of the illustrative sample, showing how key gaps and recommendations are being addressed.

### 4.5. Artificial Intelligence as a Unifying Layer Across ICF Domains

Recent AI studies explore adaptive, predictive, personalized AT [77,78,79,80,81,82,83,84,85], based on the research keywords and conceptual trajectories identified in [85], it clearly emerges that—even in conditions of paraplegia or tetraplegia—intervention cannot be limited to motor impairment alone. According to the ICF framework, functioning is defined by the interaction of multiple health components, and disability may involve not only mobility, but also communication, intellectual and cognitive functions, autonomy, emotional regulation, and social participation. From an ICF perspective, focusing exclusively on bodily motor functions represents a partial and incomplete interpretation of health.

Within the ICF model, paraplegia and tetraplegia are therefore understood as multidimensional conditions, in which impairments, activity limitations, and participation restrictions coexist across several ICF domains. Communication, cognition, and autonomy may become as critical as mobility in determining overall functioning and quality of life. The ICF explicitly requires that assistive strategies address this plurality of domains, recognizing disability as the outcome of interactions between the individual, technologies, and the environment.

In this strongly ICF-oriented context, assistive technologies—and particularly AI-based systems—operate as integrative enablers across ICF components. AI functions as a connective layer within the ICF framework, linking bodily functions, activities, participation, and environmental factors. Through ICF-aligned personalized assessment and adaptive rehabilitation, AI supports mobility while simultaneously enhancing communication, cognitive processing, and independent decision-making [77,78,79,80,81] (enabling individualized and context-aware interventions).

In practical applications, robotics, computer vision, and deep learning directly operationalize the ICF by enabling alternative communication pathways, environmental control, and task execution within daily activities, thereby reinforcing participation as defined by the ICF [82,83,84,85,86]. Complementary AI-driven systems—including emotion recognition, cognitive assistance, navigation support, and brain–computer interfaces—further extend ICF-based interventions to intellectual, communicative, and social domains, reinforcing autonomy and participation within an explicitly ICF-consistent framework [87,88,89].

Clinical and monitoring technologies, such as adaptive prosthetics, EEG-based analytics, and AIoT-enabled systems, demonstrate how precision and context-aware support can strengthen rehabilitation and long-term care when designed according to ICF principles [90,91,92,93,94] including studies from adjacent clinical domains. Although several of these approaches originate from adjacent populations—such as stroke survivors, older adults, or individuals with communication problems—the research strategies and keywords highlighted in [85,90] reflect ICF-compatible concepts of personalization, adaptive interaction, and user-centered design that are directly transferable to paraplegia and tetraplegia.

Overall, when repeatedly framed within the ICF, these examples emphasize that AI-driven assistive technologies do not merely compensate for motor loss, but actively implement the ICF vision of functioning. By systematically connecting mobility, communication, cognition, autonomy, and social participation within a unified technological ecosystem, AI embodies the ICF as both a conceptual and operational framework, enabling inclusion, independence, and empowerment even in the presence of severe motor impairment.

### 4.6. Limitations

This narrative review was primarily designed to synthesize findings from review studies, aiming to provide a comprehensive and conceptually integrated overview of the field. To address common limitations associated with narrative syntheses, several mitigating strategies were applied.

First, available empirical studies specifically focused on the field—including randomized controlled trials (RCTs), clinical trials, and observational studies—were systematically retrieved and included. These studies complement broader evidence syntheses, offering insights into emerging trends and methodological gaps highlighted in the reviewed literature.

Second, using a dedicated checklist [21] and a consolidated study-qualification method [22], the narrative approach allowed integration of diverse forms of evidence while maintaining conceptual depth and clinical relevance [19]. These elements were combined with review-of-reviews methodologies [20] and differential discussion approaches incorporating innovative studies and policy documents, providing a structured way to compare evidence, identify recurring trends, and highlight gaps or areas where knowledge remains limited.

Third, the review’s focus on assistive and medical applications—rather than purely technical developments—may have excluded some engineering-oriented studies. However, this ensured alignment with clinical and functional priorities, enhancing translational relevance for rehabilitation and patient-centered care.

Fourth, the review integrates a translational dimension by incorporating international policy documents and frameworks—such as WHO-GATE, WHO-MTS-SCI-E, ICF, the Durable Medical Equipment Guide for Persons with SCI or Dysfunction (ASIA, 2022), Assistive Technology Guidance MHRA in UK, European Accessibility Act (EAA, Dir. 2019/882), Canada’s Accessibility Standards (CAN/ASC-EN 301 549:2024), and Integrated Chinese & Western Medicine Rehabilitation Guidelines for SCI (C-CPG)—to contextualize gaps and recommendations within global strategies for AT innovation and equitable access.

Fifth, while much of the analysis centers on AI-driven approaches, complementary ATs were also considered to capture the convergence of intelligent systems, robotics, and user-centered design, supporting a holistic understanding of integrated solutions for individuals with tetraplegia.

### 4.7. Implication for Clinical Practice and Future Research

Assistive technologies (ATs) are essential tools to promote autonomy, participation, and quality of life in individuals with tetraplegia. Evidence from clinical trials, pilot studies, and comprehensive reviews demonstrates that interventions that are personalized, interoperable, and human-centered, integrating neurotechnological, robotic, digital, and psychosocial innovations, can produce meaningful improvements in functional outcomes [34,35,36,37,38,39,40,41,42,43,44,55,56,57,58,59,60,61,62,63,64,65,66,67,68].

Devices such as powered exoskeletons, BCI-controlled robotics, soft robotic gloves, and adaptive interfaces have demonstrated safety, feasibility, and functional benefits, particularly when developed through participatory design and co-evaluation methodologies, ensuring user engagement, adherence, and sustained use [55,56,60,61,63]. For example, Lang et al. [55] confirmed the TWIICE powered exoskeleton’s safety and usability, while Bellicha et al. [56] highlighted the feasibility of BCI-assisted robotic devices for home-based mobility and object manipulation. Similarly, Osuagwu et al. [60] and Cappello et al. [63] demonstrated improvements in hand function through soft robotic gloves, emphasizing real-world applicability.

#### 4.7.1. Psychosocial and User-Centered Dimensions

Psychosocial and user-centered aspects—including emotional adaptation, motivation, sense of agency, and user acceptance—are increasingly recognized as critical determinants of assistive technology (AT) success, yet they remain underexplored in both clinical and technological research [34,37,40,62]. Addressing these dimensions is essential, as long-term adherence, satisfaction, and engagement depend not only on device functionality but also on how users perceive control, autonomy, and relevance to daily life.

Evidence suggests that participatory design and co-creation methodologies, which actively involve end-users in developing and refining AT, significantly enhance acceptance and functional outcomes. For instance, studies on powered exoskeletons demonstrate that individuals with spinal cord injury report higher motivation, confidence, and willingness to continue therapy when interventions are tailored through iterative user feedback [55,61]. Similarly, intraoral tongue-controlled interfaces and multimodal adaptive devices show that integrating user preferences into the design improves usability, task performance, and perceived autonomy [62,64].

Beyond functional performance, psychosocial factors such as self-efficacy, mental well-being, and social participation are pivotal in determining the real-world impact of AT. Incorporating structured psychosocial evaluation—through validated scales for quality of life, emotional adaptation, and participation—can help bridge the gap between laboratory efficacy and meaningful daily-life outcomes [60,63,65]. This holistic approach ensures that AT interventions support not only motor recovery but also cognitive, emotional, and social dimensions of rehabilitation.

Future research should prioritize systematic assessment of psychosocial outcomes alongside technical performance, integrating these measures into both clinical trials and translational studies. By embedding psychosocial and user-centered evaluation into the development and implementation of AT, researchers and clinicians can better promote adherence, enhance quality of life, and foster sustainable, inclusive rehabilitation strategies for individuals with tetraplegia [34,37,55,56,57,58,59,60,61,62,63,64,65,66,67,68].

#### 4.7.2. Inequities in Access and Contextual Variability

Persistent inequities in access, socioeconomic barriers, and international disparities continue to constrain the adoption and effective use of assistive technologies (AT) [34,42,44]. Advanced devices—such as powered exoskeletons, BCI-controlled robotics, and adaptive interfaces—are predominantly concentrated in high-resource settings, leaving substantial gaps in low- and middle-income countries where infrastructure, funding, and specialized clinical expertise may be limited [42,44].

These disparities not only affect availability but also impact user training, long-term support, and integration into daily life. Socioeconomic constraints, including high device costs and limited reimbursement schemes, further exacerbate the accessibility gap, potentially reducing adherence and limiting the overall impact of AT interventions [34,43].

Future research should explicitly address these inequities by exploring cost-effective designs, modular and scalable technologies, and adaptable implementation strategies suitable for diverse resource contexts. Developing solutions that are not only technologically advanced but also affordable and maintainable can expand AT reach and usability across different populations [43,44].

Policymakers, clinicians, researchers, and designers must collaborate to establish inclusive infrastructures, equitable reimbursement models, and training programs that support widespread AT dissemination. Such coordinated strategies are essential to ensure that technological innovation translates into real-world benefits, promoting autonomy, participation, and quality of life for individuals with tetraplegia globally [42,43,44].

#### 4.7.3. Integration of the ICF Framework

The International Classification of Functioning, Disability and Health (ICF) [95] offers a comprehensive framework to organize and interpret the impact of interventions across multiple functional domains, including body functions, activities, participation, and environmental factors. Although most current studies focus on motor recovery, neurotechnological performance, or psychosocial outcomes [34,35,36,37,38,39,40,41,42,43,44,55,56,57,58,59,60,61,62,63,64,65,66,67,68], the ICF could potentially serve as a useful framework to structure and compare these effects across interventions.

For example, Neural and Regenerative Interfaces—such as brain–computer interfaces, functional electrical stimulation, or neuroplasticity-targeted devices—might be mapped to ICF domains by linking changes in neural control and muscle activation to “body functions,” while improvements in task execution could relate to “activities,” and increased independence in daily life could be considered under “participation” [55,59,65]. Similarly, Motor and Biomechanical Assistive Systems, including exoskeletons, robotic arms, or adaptive orthoses, could be evaluated not only for their biomechanical efficacy but also for their potential impact on mobility-related participation and social or occupational engagement [56,60,63].

Digital and Adaptive Rehabilitation Ecosystems—such as tele-rehabilitation, VR-based training, or mobile health platforms—may influence multiple ICF domains simultaneously, enhancing “activities” through skill practice, supporting “participation” via community-based rehabilitation, and modifying “environmental factors” by facilitating remote access or adaptive interfaces [25,42,57,58]. Finally, Psychosocial and Integrative Frameworks—including interventions targeting user engagement, emotional adaptation, and self-efficacy—could address psychosocial dimensions aligned with both “participation” and “environmental factors,” highlighting the importance of context, social support, and inclusive design [34,37,40,62,64].

By cautiously applying the ICF framework, researchers and clinicians might better capture the multidimensional effects of AT, facilitating cross-study comparisons, identifying gaps in unaddressed functional domains, and supporting the translation of research into real-world, patient-centered rehabilitation strategies [34,35,36,37,38,39,40,41,42,43,44,55,56,57,58,59,60,61,62,63,64,65,66,67,68].

#### 4.7.4. AI and Data-Driven Enhancements

Artificial intelligence (AI) offers promising avenues to strengthen assistive technology (AT) ecosystems by enabling personalized assessment, predictive monitoring, adaptive rehabilitation, and context-aware interaction. Recent literature highlights how machine learning, deep learning, and AI-driven frameworks can support rehabilitation workflows, decision support, and assistive control systems that adapt to individual needs and environments [77,78,79,80,81,85,89]. For example, AI-based motion analysis and pattern recognition systems can tailor therapy to user performance, while generative models and predictive algorithms can anticipate functional needs and optimize device parameters in real time [80,81,85].

Beyond motor control, AI can enhance communication, environmental interaction, and accessibility by powering intelligent interfaces that respond to user intent and contextual cues, including natural language, gaze, and facial expression recognition systems [82,87,89]. These capabilities expand AT from purely mechanical compensation toward interactive, adaptive ecosystems that support autonomy across daily life situations.

However, the deployment of AI in AT must be guided by robust ethical frameworks and human-centered design principles to ensure equity, transparency, and respect for user autonomy. Issues such as data privacy, explainability, algorithmic fairness, and informed consent are essential to prevent biases and unintended harm, particularly when models are trained on limited or non-representative datasets [85]. AI should therefore complement—not replace—the user’s agency and psychosocial support structures, embedding intelligent systems within broader patterns of rehabilitation that honor personal values and lived experience.

#### 4.7.5. Future Directions for Research and Clinical Practice

To advance the field, future research should focus on the following priorities:

1. Longitudinal evaluation of clinical, functional, and psychosocial outcomes to assess sustained effectiveness and real-world adoption [34,38,55,60,61].
2. Interoperable and modular architectures that integrate neurotechnological, robotic, digital, and psychosocial components into cohesive assistive ecosystems [36,39,42,57,58].
3. Systematic integration of psychosocial measures, including emotional adaptation, motivation, quality of life, and social participation, embedded in clinical and technological studies [35,37,40,62,64].
4. Explicit use of the ICF framework, connecting interventions to body functions, activities, participation, and environmental factors for a holistic evaluation of rehabilitation outcomes [36,37,41].
5. Addressing inequities and global disparities, designing cost-effective, adaptable solutions for diverse socio-economic and cultural contexts [42,44].
6. Ethical and data governance considerations, especially for AI-driven or invasive interventions, ensuring transparency, autonomy, and protection of sensitive information [37,40,62,65].

By implementing these strategies, future AT research and practice can translate technological innovation into tangible improvements in independence, participation, and quality of life for individuals with tetraplegia. This approach ensures that AT interventions are scientifically rigorous, ethically grounded, socially equitable, and fully user-centered, integrating physical, cognitive, and psychosocial dimensions into a holistic, interoperable, and human-focused rehabilitation ecosystem.

## 5. Conclusions

ATs for tetraplegia have transformed over the past seven decades, evolving from mechanical aids to advanced neurotechnological and digital systems. This narrative synthesis highlights both the remarkable progress and the persistent gaps in evidence, methodology, and access. Current research demonstrates rapid innovation across robotics, neurointerfaces, digital rehabilitation, and psychosocial support, yet fragmentation, limited interoperability, ethical uncertainties, and inequities remain significant barriers. Recent clinical studies and participatory approaches show that adaptive, user-centered, and AI-enabled systems can enhance autonomy, engagement, and functional outcomes. International and national guidelines increasingly support standardization, inclusivity, and rights-based approaches, although implementation is uneven. Artificial intelligence emerges as a powerful enabler, fostering personalization, real-time adaptation, and holistic care within the International Classification of Functioning framework. Moving forward, coordinated strategies are essential to integrate research, clinical practice, and policy, with priority on ethical AI, interoperability, standardized outcomes, and equitable access, ensuring sustainable, person-centered innovation that maximizes autonomy, participation, and quality of life for individuals with tetraplegia.

## Figures and Tables

**Table 1:** Focus Areas, Clinical Terms, and Assistive Technology Categories for Tetraplegia.

Focus Area	Clinical/Functional Terms	Assistive Technology/Device/Product Terms	Notes	Functional Domain/Outcome Focus
General-Purpose Search/Boolean Query	(tetraplegia OR tetraplegic* OR quadriplegia OR quadriplegic* OR “spinal cord injury” OR “SCI”)	(“Assistive tech*” OR “Assistive dev*” OR “Assistive device*” OR “Assistive product*”)	Core search string to capture all relevant literature across domains; provides a replicable foundation for ongoing and future research in assistive technology for tetraplegia	Supports identification of relevant studies and informs future developments across functional domains
Mobility and Upper-Limb Function	“tetraplegia”, “quadriplegia”, “upper limb function”, “arm/hand function”, “mobility impairment”	“wheelchair”, “powered exoskeleton”, “standing device”, “prosthetic”, “robotic assistance”, “assistive technology”, “assistive device*”, “assistive product*”	Devices supporting movement and daily tasks	Mobility, upper-limb function, independence in daily activities
Communication	“communication impairment”, “speech difficulty”, “social participation”	“speech-generating device”, “eye-tracking system”, “head-controlled interface”, “brain-computer interface”, “AAC device”, “assistive technology”, “assistive device*”, “assistive product*”	Enhances interaction, education, work, and social life	Communication, social engagement, participation
Daily Living and Environmental Control	“self-care”, “activities of daily living”, “independence”	“adaptive tools for eating/dressing”, “environmental control systems”, “home automation”, “assistive technology”, “assistive device*”, “assistive product*”	Supports autonomy at home and in daily routines	Daily living activities, autonomy, quality of life
Psychosocial and Quality of Life	“well-being”, “mental health”, “social inclusion”, “participation”	“assistive technology”, “assistive device*”, “assistive product*”, “telehealth platform”, “digital mental health tool”	Includes both device-based and non-device interventions, providing context for integrated assistive technology use”	Quality of life, mental health, social participation

**Table 2:** Inclusion and Exclusion Criteria.

Criterion	Definition	Inclusion	Exclusion	Notes
Study Type	Type of publication	Reviews (systematic, scoping, narrative, meta-analytic, perspective)	Original research, case reports, technical notes	Ensures high-level evidence
Relevance	Clinical, technological, or psychosocial application	AT interventions with functional outcomes for tetraplegia	Purely technical or computational studies without clinical relevance	Focus on real-world impact
Temporal Scope	Publication period	Up to 30 October 2025	Older publications	Updated post-pandemic coverage
Language	Language of publication	English	Non-English	Accessibility and reproducibility
Focus	Main content	Functional, clinical, or psychosocial outcomes	Technical-only methods	Maintains focus on narrative synthesis

**Table 3:** Comparative Trends on Tetraplegia-Related and General AT Research (1953–2025).

Research Focus	Total Publications (1953–2025)	Reviews (Systematic, Non-Systematic, Meta-Analysis)	Publications Last 10 Years	Publications Last 5 Years
Tetraplegia&SCI + AT	544	74 (13.6%)	343 (63%)	187 (34%)
AT (general)	8650	1288 (14.9%)	5942 (68.7%)	3789 (43.8%)

**Table 4:** Overview of included studies on assistive technologies in tetraplegia.

Ref.	Brief Description	Focus	AT Application
Núñez Sardinha et al. (2025) [24]	Systematic review on the effectiveness and user acceptance of assistive technologies for people with tetraplegia.	The study addresses how usability, emotional connection, and sense of control shape long-term engagement with assistive devices. It underscores that real-world success depends on psychosocial adaptation as much as on mechanical reliability.	ATs are presented as co-created tools that expand autonomy and dignity, aligning device functionality with the user’s identity and self-efficacy.
Mota et al. (2025) [25]	Scoping review on mobile health applications supporting rehabilitation in spinal cord injury.	The authors map the emergence of mobile platforms as intermediaries between clinical supervision and self-managed recovery. They focus on usability, feedback quality, and digital literacy as barriers and opportunities.	mHealth tools are reframed as personalized, adaptive supports that can foster motivation and continuous engagement in remote rehabilitation routines.
Elliott et al. (2025) [26]	International recommendations on sexual and reproductive health in neurological disorders, including SCI.	The review highlights how sexuality remains an under-integrated component of neurological rehabilitation. It connects sexual function with psychological health and social participation.	Assistive solutions such as positioning devices or sensory feedback systems are described as mediators of intimacy, restoring agency and body image beyond medical functionality.
Kang et al. (2025) [27]	Systematic review of usability testing for manual wheelchairs in individuals with SCI.	The focus lies on optimizing wheelchair design to prevent shoulder injuries and enhance mobility in daily life. Usability testing emerges as a human-centered practice balancing biomechanical data and lived experience.	Wheelchair ergonomics and adjustability are portrayed as adaptive systems co-evolving with the user’s physical changes and activity patterns.
Stieglitz et al. (2025) [28]	Comparative review of electrical stimulation paradigms after central paralysis and amputation.	The study explores how neurostimulation strategies differ in restoring function, emphasizing tailored parameters and plasticity-based models.	Electrical stimulation technologies are proposed as modular frameworks for neurorehabilitation, adaptable to residual function and patient-specific goals.
De Pastina et al. (2025) [30]	Systematic review on remapping of peripersonal space after stroke, SCI, and amputation.	The authors examine how injury alters body-space representation, affecting interaction with tools and environments.	ATs are envisioned as extensions of peripersonal space, offering sensory feedback and spatial recalibration to support embodiment and movement control.
Mitchell et al. (2025) [31]	Metasynthesis on workplace experiences with assistive technologies among people with SCI.	The study addresses inclusion, identity, and agency in professional contexts, identifying psychosocial and organizational enablers of AT adoption.	ATs at work are interpreted as socio-technical mediators enabling participation and autonomy through customized adaptive interfaces and environmental adjustments.
Sawyer et al. (2024) [32]	Perspective paper advocating for implanted brain–computer interfaces (BCIs) in severe quadriplegia.	The focus is on bridging human intention and external control through invasive neurotechnologies, addressing ethical, safety, and usability challenges.	BCIs are presented as transformative tools for restoring communication, environmental control, and even affective interaction in individuals with complete paralysis.
Arellano & Vega (2024) [33]	Experimental synthesis on the biomechanical interplay between upper and lower limbs in gait rehabilitation.	The study explores compensatory mechanisms and inter-limb coordination, suggesting new training paradigms leveraging arm–leg synergies.	Robotic and sensorized systems are proposed to integrate upper-limb effort into locomotor rehabilitation, promoting whole-body engagement in therapy.
An et al. (2023) [34]	Review on design requirements for universally accessible upper-body exercise equipment in people with SCI.	The paper examines inclusion and ergonomics in public and therapeutic environments, advocating for universal design principles.	Adaptive fitness equipment is conceptualized as both rehabilitative and social tools, encouraging physical activity and participation across disability levels.
Morone et al. (2023) [35]	Narrative review of reviews on the development and integration of ATs in the healthcare system.	The study highlights evolution trends, regulatory barriers, and integration bottlenecks across assistive domains.	ATs are seen as evolving ecosystems bridging clinical innovation, user co-design, and digital health infrastructures.
Alazzam et al. (2023) [36]	Review on metabolic rate measurement after SCI.	Although physiologically oriented, it underlines the importance of personalized energy expenditure models in rehabilitation planning.	Wearable and sensor-based ATs are identified as potential tools for dynamic metabolic monitoring, enabling precision in exercise and nutrition management.
Gurung et al. (2023) [37]	Scoping review on sociostructural and environmental factors influencing health and quality of life in SCI.	The study contextualizes rehabilitation within social ecology, emphasizing the role of environment, accessibility, and policy.	ATs are framed as instruments of social inclusion that mitigate structural barriers through adaptive housing, mobility aids, and smart-environment interfaces.
Fallahzadeh Abarghuei & Karimi (2022) [38]	Systematic review on the effects of lower-limb orthoses in SCI using the ICF framework.	The paper integrates functional, psychological, and social outcomes of orthotic use, redefining their role beyond mechanical support.	Orthoses are discussed as dynamic enablers of upright posture, mobility, and confidence, connecting biomechanics with psychosocial rehabilitation.
Pirrera et al. (2022) [39]	Review on the development and dissemination of tongue barbell piercing as a control interface for quadriplegic users.	The study rethinks bodily interfaces by transforming a symbolic object into a communication and control tool.	The tongue piercing system exemplifies how cultural adaptation and user creativity can redefine the human–machine boundary in assistive design.
Colucci et al. (2022) [40]	Review on BCI-controlled exoskeletons for neurorehabilitation.	The focus lies on readiness, ethical adoption, and translational challenges of hybrid brain–machine–body systems.	Exoskeletons integrated with BCIs are conceptualized as neuroprosthetic extensions enabling volitional control and sensorimotor feedback restoration.
Onate-Figuérez et al. (2023) [41]	Systematic review and meta-analysis on fatigue in people with SCI.	The paper examines multifactorial fatigue as a barrier to participation and recovery, linking physiological and psychosocial dimensions.	ATs and wearables are proposed as tools for continuous monitoring and adaptive workload management, informing individualized rehabilitation plans.
Vibhuti et al. (2023) [42]	Systematic review on virtual reality therapy for neuromotor rehabilitation in home environments.	The study explores immersive VR as a means to enhance engagement, repetition, and embodiment in rehabilitation.	VR platforms are presented as therapeutic ecosystems enabling home-based, gamified neurorehabilitation that supports autonomy and continuity.
Khalid et al. (2023) [43]	Systematic review of robotic assistive and rehabilitation devices for upper-limb motor recovery.	The authors analyze performance outcomes and human–robot interaction dynamics across robotic modalities.	Robotics are positioned as catalysts for motor relearning, integrating adaptive feedback and task-oriented control for personalized neurorehabilitation.
Plaza et al. (2023) [44]	Review of wearable lower-limb rehabilitation exoskeletons analyzing adaptability and versatility.	The study highlights modularity, comfort, and user-centered calibration as determinants of clinical success.	Wearable exoskeletons are conceived as flexible prosthetic supports enhancing gait training and participation across stages

**Table 5:** Conceptual domains of categorization for the reviews on assistive technology studies in tetraplegia.

Domain	Description	Included Studies (References)
Neural and Regenerative Interfaces	This domain encompasses studies exploring the interface between neural repair, neuroplasticity, and assistive control systems. Research in this area investigates brain–computer interfaces, neuromodulation, and regenerative strategies for functional recovery after spinal cord injury. These approaches aim to restore communication between brain and body through direct or indirect neural pathways.	[28,30,32,40]
Motor and Biomechanical Assistive Systems	Studies in this domain address physical mobility, motor control, and ergonomic optimization. They include investigations on robotic exoskeletons, orthoses, and adaptive exercise equipment designed to enhance motor performance and reduce musculoskeletal strain. The common goal is to improve independence through biomechanical synergy and human–device co-adaptation.	[27,33,34,38,43,44]
Digital and Adaptive Rehabilitation Ecosystems	This domain integrates mobile health, virtual reality, and data-driven rehabilitation platforms. It highlights the transition toward smart, connected assistive systems capable of remote monitoring and personalized feedback. These studies underline the importance of interoperability, user engagement, and ecological validity in digital rehabilitation.	[25,35,42]
Psychosocial and Integrative Frameworks	The psychosocial domain examines user experience, social participation, and environmental adaptation in assistive technology use. It includes both empirical and conceptual works emphasizing inclusivity, autonomy, and mental well-being. Together, these studies frame assistive technology as a socio-technical construct rather than a purely mechanical aid.	[24,26,31,36,37,39,41]

**Table 6:** Emerging cross-cutting gaps and recommendations in assistive technology for tetraplegia.

Number	Identified Gaps	Emerging Directions/Recommendations	Potential Role of AI
1	Limited methodological robustness—Many studies rely on small samples, short follow-ups, and heterogeneous outcome measures, reducing comparability and clinical generalization [25,32,40,42].	Promote multicenter longitudinal trials with standardized protocols, integrating quantitative and qualitative metrics for usability, safety, and quality of life. Encourage open datasets for benchmarking.	AI can enable adaptive trial designs, automate outcome analysis, and detect longitudinal trends from multimodal data streams.
2	Fragmented technological ecosystems—Neural, mechanical, and digital systems remain poorly integrated, limiting seamless operation and data exchange [27,35,44].	Develop interoperable architectures and shared communication standards that allow modular integration across devices and therapeutic settings.	AI can act as an interoperability layer, harmonizing inputs across sensors, interfaces, and contexts to support adaptive, personalized assistance.
3	Low user acceptance and discontinuity in use—Many devices fail to align with the evolving needs, preferences, and psychosocial realities of users [24,31].	Implement co-design methodologies and participatory evaluation frameworks to ensure that technologies evolve with user feedback and daily-life demands.	AI-driven personalization can dynamically adapt device settings and feedback loops based on user behavior and emotional or physiological states.
4	Ethical and regulatory uncertainty—Invasive systems (e.g., implanted BCIs, tongue interfaces) raise concerns about autonomy, consent, and privacy [32,39].	Establish adaptive ethical guidelines and inclusive governance models ensuring transparency, data protection, and shared decision-making.	AI systems must embed explainability and privacy-by-design principles to safeguard user dignity and informed control.
5	Inequitable access and contextual variability—Socioeconomic, geographic, and environmental barriers hinder adoption and sustainability [31,37].	Foster policy frameworks and reimbursement models that support equitable access, local adaptation, and long-term maintenance of assistive technologies.	AI can support scalable remote monitoring, resource allocation, and context-sensitive personalization for diverse environments.
6	Insufficient integration of psychosocial rehabilitation—Emotional well-being, sexuality, and social participation are often peripheral in AT design [26,37,41].	Incorporate holistic outcome measures and interdisciplinary teams addressing mental health, sexuality, and community reintegration alongside motor recovery.	AI-based conversational agents and adaptive interfaces could enhance motivation, emotional support, and social inclusion.

**Table 7:** Mapping of recent studies on AT for tetraplegia to identified gaps and recommendations (2016–2025).

Study	Gap/Recommendation	Focus/Contribution
Lang et al., 2025 [55]	1, 3	Safety and feasibility of powered exoskeletons; addresses methodological robustness and user acceptance.
Bellicha et al., 2025 [56]	2, 3	BCI-controlled assistive robotics for long-term home use; tackles ecosystem integration and co-design for usability.
de Crignis et al., 2023 [57]	1, 3	Robotic arm training with AR; improves methodological rigor and patient-centered adaptation.
Rubin et al., 2023 [58]	1, 4	Interim safety of BrainGate neural interface; addresses trial robustness and ethical/regulatory considerations.
Ting et al., 2021 [59]	2, 3	Neural decoding for tetraplegia; supports interoperability and personalized assistive control.
Osuagwu et al., 2020 [60]	1, 3, 6	Soft robotic hand glove for home rehab; enhances clinical evidence, user engagement, and psychosocial outcomes.
Manns et al., 2019 [61]	3	User perspectives on powered exoskeletons; informs acceptance and real-world usability.
Kong et al., 2019 [62]	2, 3	Tongue-controlled interface; contributes to system integration and personalization.
Cappello et al., 2018 [63]	1, 3, 6	Soft robotic glove; improves methodological rigor, user-centered design, and psychosocial rehabilitation.
Sahadat et al., 2018 [64]	2, 3	Multimodal PC access; supports ecosystem integration and adaptive user interfaces.
Ajiboye et al., 2017 [65]	1, 3	Brain-controlled muscle stimulation; strengthens clinical trial evidence and functional restoration.
Pandarinath et al., 2017 [66]	1, 3	High-performance BCI for communication; enhances methodological robustness and user-centered outcomes.
Osuagwu et al., 2016 [67]	1, 3, 6	BCI and FES hand rehab; addresses methodological rigor, acceptance, and psychosocial integration.
Downey et al., 2016 [68]	2, 3	Blended BCI and autonomous robotics; improves integration and user-centered neuroprosthetic control.
Martin-Lemoyne et al., 2016 [69]	3	Mobility assistance dog study; highlights user acceptance and ergonomic adaptation for wheelchair users.

**Table 8:** Illustrative sample of international and national guidelines on assistive technologies and tetraplegia, exemplifying how key gaps and recommendations are being addressed.

	Organization/Country	Title/Year	Main Focus	Gaps Addressed
1	World Health Organization (WHO)	Minimum Technical Standards and Recommendations for Spinal Cord Injury Management in Emergencies (WHO-MTS-SCI-E)	Defines essential requirements for coordinated care, rehabilitation, and use of AT in emergency contexts	1, 4, 5
2	World Health Organization (WHO)	Global Cooperation on Assistive Technology (GATE) Initiative	Promotes equitable access, affordability, and governance of AT globally	1, 4, 5
3	American Spinal Injury Association (ASIA, USA)	Durable Medical Equipment Guide for Persons with Spinal Cord Injury or Dysfunction (2022 Edition)	Guidance on device selection, training, and reimbursement for SCI and tetraplegia	1, 3
4	Medicines and Healthcare products Regulatory Agency (MHRA, UK)	Assistive Technology: Definition and Safe Use (2025)	Ensures technical safety, interoperability, and ethical use of AT	2, 4
5	European Union	European Accessibility Act (Directive (EU) 2019/882)	Establishes accessibility standards for AT products and services	2, 5
6	Accessibility Standards Canada	CAN/ASC-EN 301 549:2024	Implements user-centered design and inclusive procurement for AT	3, 5
7	China (National Health Commission)	Clinical Practice Guidelines of Integrated Chinese and Western Medicine Rehabilitation for Spinal Cord Injury	Integrates medical and social rehabilitation for equitable access to AT in tetraplegia	5, 6

## Data Availability

No new data were created or analyzed in this study.

## Supplementary Materials

The following supporting information can be downloaded at: https://www.mdpi.com/article/10.3390/healthcare14020274/s1, Section S1: Independent Quality Assessment of Included Studies; Table S1: Mean scores assigned to each study (the study is anonymized).
