# Supplementary material for: Innovative Assistive Technologies for Tetraplegia: A Narrative Review of Systematic and Emerging Evidence
Source: Healthcare (Basel). 2026 Jan 21;14(2):274. doi: 10.3390/healthcare14020274 (PMC12840652; doi:10.3390/healthcare14020274)
Supplement: Supplementary file 1 [file healthcare-14-00274-s001.zip › healthcare-4046526-supplementary.pdf]

SUPPLEMENTARY FILE

Review

Innovative Assistive Technologies for Tetraplegia: A Narrative Review of Systematic and Emerging Evidence

Lorenzo Desideri [1], Regina Gregori Grgič [1], Antonia Pirrera [2] and Daniele Giansanti [2,*]

[1] Digital Psychology Lab, Sigmund Freud University, 20143 Milano, Italy; l.desideri@milano-sfu.it (L.D.); r.gregori@milano-sfu.it (R.G.G.)
[2] National Center IATIS, Istituto Superiore di Sanità-Roma, 00161 Roma, Italy; antonia.pirrera@iss.it
* Correspondence: daniele.giansanti@iss.it or gianslele@gmail.com

Section S1 – Independent Quality Assessment of Included Studies

All studies ultimately included in the narrative synthesis underwent rigorous, independent quality assessment using the six-parameter scale (N1–N6) described in the methods. Each study was scored independently by two reviewers: N1–N5 on a 1–5 scale (1 = Poor, 5 = Excellent), allowing non-integer (decimal) values, and N6 (disclosure of conflicts of interest) as Yes/No. For all 83 full-text studies, N6 was scored as Yes by both reviewers. Only studies meeting the pre-defined thresholds for N1–N5 for both reviewers were included in the narrative synthesis.

The table s.1 below presents anonymized mean scores for N1–N5 for each reviewer for the 20 included studies. Studies are listed in an anonymized order, independent of the order in which they appear in the main narrative review.

Of the 83 full-text studies initially screened, 62 were excluded after quality assessment: 55 failed to meet the N1–N5 thresholds for both reviewers, and 7 failed to meet the threshold for one reviewer. These studies were therefore not included in the narrative synthesis.

One study, reference [29], was considered borderline in relation to the focus of the invetigation. Due to its relevance and impact, it was cited in the review but not included in the detailed analysis. This resulted in a total of 21 studies being selected for discussion, with 20 included in the formal analysis.

Table S1. Mean scores assigned to each study (the study is anonymized)

Study ID	Reviewer 1 Mean (N1–N5)	Reviewer 2 Mean (N1–N5)	N6 (Disclosure)
1	4.1	4.0	Yes
2	4.3	4.2	Yes
3	4.0	4.2	Yes
4	4.4	4.1	Yes
5	4.0	4.1	Yes
6	3.8	4.0	Yes
7	4.2	4.1	Yes
8	4.1	4.0	Yes
9	4.3	4.1	Yes
10	4.0	4.2	Yes

11	3.9	4.0	Yes
12	4.2	4.1	Yes
13	4.1	4.0	Yes
14	4.3	4.2	Yes
15	4.1	4.0	Yes
16	4.0	3.9	Yes
17	4.2	4.1	Yes
18	4.3	4.0	Yes
19	4.1	4.2	Yes
20	3.9	4.0	Yes
